# Commentary on exploring potential plasma drug targets for cholelithiasis through multiancestry mendelian randomization

**DOI:** 10.1097/JS9.0000000000003286

**Published:** 2025-08-25

**Authors:** Ji Pan, Yupeng Liu

**Affiliations:** a Department of Pharmacy, Xinchang County People’s Hospital, Shaoxing, Zhejiang, People’s Republic of China; b Department of Clinical Pharmacology, Affiliated Hangzhou First People’s Hospital, School of Medicine, Westlake University, Hangzhou, People’s Republic of China; c Key Laboratory of Clinical Cancer Pharmacology and Toxicology Research of Zhejiang Province, Hangzhou First People’s Hospital, Hangzhou, People’s Republic of China

*Dear Editor,*

We read with great interest the recent article exploring potential drug targets for gallstone disease using plasma proteome-based Mendelian randomization (MR) in both European and East Asian populations^[1]^. This comprehensive study provides valuable insights into the genetic architecture and therapeutic potential of several protein targets. While we appreciate the innovative approach taken, we would like to offer some alternative perspectives on the study.

A major limitation lies in the substantial imbalance between the European and East Asian cohorts in terms of proteomic and GWAS data depth and quality. The primary proteomic dataset for Europeans was derived from the deCODE study and included 4907 plasma proteins, whereas the corresponding East Asian data, drawn from the UKB-PPP cohort, covered only 2,941 proteins. This discrepancy may hinder the detection of statistically robust associations in the East Asian population and casts doubt on cross-population comparisons.

Despite the use of multiple sensitivity analyses including Steiger filtering, Bayesian colocalization, and reverse MR the study engages in speculative interpretations without sufficient empirical support. For instance, the proposed involvement of FKBP52 in FXR regulation and bile acid metabolism is mechanistically plausible but lacks confirmation via direct protein-protein interactions (PPIs) in databases such as STRING. Similarly, the link between NOE1, a gene primarily associated with neural crest development, and gastrointestinal phenotypes is currently not backed by adequate functional evidence to justify its consideration as a therapeutic target.

The exclusive focus on cis-pQTLs assumes that protein levels are reflected in a tissue-agnostic manner. However, circulating plasma levels may not reliably represent concentrations in key tissues such as the liver or biliary tract, which are central to gallstone pathogenesis. Prior research has shown that protein expression in liver tissue does not always correspond with plasma levels^[2]^, highlighting the need for caution when drawing conclusions about causality based solely on blood biomarkers. Moreover, MR models generally rely on the assumption of linear and monotonic exposure-outcome relationships, yet the biological effects of proteins such as FGFR4 or UGT1A6 may be dose-dependent, tissue-specific, or modulated by metabolic context. These complexities are rarely accounted for in MR studies but are essential when aiming for translational drug development.

While the authors rightly emphasize ancestry-specific drug targets, they do not address intra-population heterogeneity, such as differences in sex, age, or metabolic comorbidities, which may influence both disease risk and therapeutic responses. Additionally, East Asian populations encompass diverse ethnic groups with distinct genetic backgrounds. Treating them as a homogeneous cohort may obscure important substructure and lead to oversimplified conclusions.

In closing, we sincerely commend Liu and colleagues for their important work in identifying potential protein targets for gallstone disease. This study contributes significantly to our understanding of the relationship between circulating proteins and disease risk. Nonetheless, we raise several critical considerations that may help refine and strengthen future research in this area. Integrating tissue-specific expression profiles, longitudinal clinical data, and experimental validation will be vital for advancing these promising findings toward clinical application. The above content has been written following the TITAN guidelines^[3]^.

## Data Availability

Not applicable.
